# Enhanced Patient-Centricity: How the Biopharmaceutical Industry Is Optimizing Patient Care through AI/ML/DL

**DOI:** 10.3390/healthcare10101997

**Published:** 2022-10-11

**Authors:** Kelly H. Zou, Jim Z. Li

**Affiliations:** Global Medical Analytics and Real-World Evidence, Viatris Inc., 1000 Mylan Blvd., Canonsburg, PA 15317, USA

**Keywords:** artificial intelligence, biopharmaceutical industry, Coronavirus Disease 2019, data science, deep learning, digital innovation, machine learning, patient-centricity, randomized controlled trials, real-world data

## Abstract

Technologies utilizing cutting-edge methodologies, including artificial intelligence (AI), machine learning (ML) and deep learning (DL), present powerful opportunities to help evaluate, predict, and improve patient outcomes by drawing insights from real-world data (RWD) generated during medical care. They played a role during and following the Coronavirus Disease 2019 (COVID-19) pandemic by helping protect healthcare providers, prioritize care for vulnerable populations, predict disease trends, and find optimal therapies. Potential applications across therapeutic areas include diagnosis, disease management and patient journey mapping. Use of fit-for-purpose datasets for ML models is seeing growth and may potentially help additional enterprises develop AI strategies. However, biopharmaceutical companies often face specific challenges, including multi-setting data, system interoperability, data governance, and patient privacy requirements. There remains a need for evolving regulatory frameworks, operating models, and data governance to enable further developments and additional research. We explore recent literature and examine the hurdles faced by researchers in the biopharmaceutical industry to fully realize the promise of AI/ML/DL for patient-centric purposes.

## 1. Introduction

The biopharmaceutical industry is increasingly realizing the potential values of artificial intelligence (AI), machine learning (ML), and deep learning (DL) to evaluate, predict, and improve patient outcomes by deriving insights from both randomized controlled trial (RCT) data and real-world data (RWD) generated from clinical or medical settings [1].

Earlier on, seminal works in AI/ML offered a historical perspective stemming from Computer Science and Information Science since the 1950s [2,3,4,5], followed by recent works on DL/AI [6,7,8]. Diverse data sources may yield useful insights through treatment pattern analysis, patient journey mapping, and longitudinal follow-ups. While classical statistical methods and tools still play an important role in healthcare analytics and regulatory pathways, data science and digital innovation are increasingly used nowadays to examine the relationships between variables. ML and DL models are increasingly used, especially for medical devices, for their ability to work with very large datasets for predictive accuracy [9,10,11,12].

Recent studies illustrate compelling applications of AI/ML/DL for diagnosis, treatment, disease management, and patient journey mapping in several non-communicable diseases, which are generally chronic diseases. As the Coronavirus Disease 2019 (COVID-19) pandemic took hold in the United States (US), there were signs that these technologies may help in infectious diseases too. 

This paper explores a selection of these studies and the hurdles that researchers in industry and academia may need to overcome to fully realize the promise of AI/ML/DL for patients. Several key abbreviations, particularly those defined by the United States’ Food and Drug Administration (FDA) or European Union’s (EU) European Medicines Agency (EMA), are listed (Table 1).

## 2. Patient-Centricity

Patient-centered care focuses on improving an individual patient’s health outcomes, not on improving a population’s health outcomes, patient-reported outcomes, nor on measuring the performance of a healthcare institution or provider [28,29]. The term “patient-centered outcomes” was included in the US federal legislation US H.R.1865—Further Consolidated Appropriations Act, 2020 [30], and was a focus of the National Academies of Sciences, Engineering, and Medicine in their reports [31].

## 3. Adoptions

### 3.1. Disease Diagnoses

Challenges in diagnoses in terms of accuracy and reliability can lead to repeated diagnoses via multiple modalities, poor choices of therapies, and consequently high-cost burdens on the healthcare system for conditions that are difficult to diagnose and lacking in pathognomonic signs and symptoms, as well as overlapping comorbid conditions, and these negative consequences can be amplified. 

**Figure 1:**
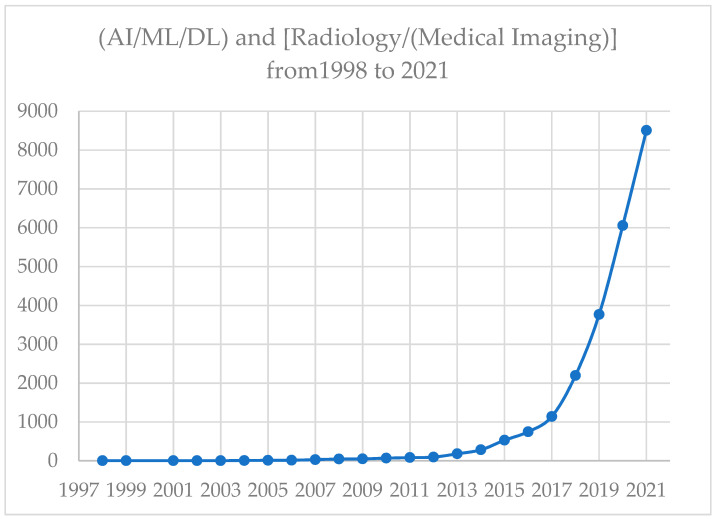
Number of articles on AI/ML/DL and Radiology (Medical Imaging) from 1998 to 2021.

Radiology, especially medical imaging, is indeed one of the fields in medicine that has had the most successful applications of AI. Over the years, it has become an essential part of medical imaging. In fact, the lead author worked in the early time of applying AI to medical imaging for several years and coauthored multiple articles, including the ones listed below. There are many publications and use-case examples of AI applications in radiology and medical imaging. Since the field is rapidly expanding and evolving, the tools and best practices to minimize biases of AI in radiology or medical imaging have been proposed [32,33,34]. For example, we obtained the numbers of PubMed-listed articles [35] by limiting publications until the end of a full year of 2021, with an understanding that 2022 is not yet a full year: using Boolean operators, and let String A alone; String R alone; Strings A and R (simply denoted as AR), where D = (“Artificial Intelligence” or “Machine Learning” or “Deep Learning”) and R = (“Radiology” or “Medical Imaging). A PubMed search of DR = D and R, yielded 4290 articles since the first article appearing in 1998 until the end of 2021. The trend based on this literature search is displayed (Figure 1). 

There are a range of ways in which AI/ML/DL can support more accurate and reliable diagnosis of conditions that can severely impair patients’ quality of life. Since big data are mostly unstructured, natural language processing of texts [36], as well as medical image analysis of CAT scans, magnetic resonance images or ultrasound images [37], can be useful. AI-based diagnostic approaches could complement physicians’ efforts, creating macro efficiencies in the healthcare system and significant quality-of-life benefits for individual patients. In Section 6.2, methodological details on the applications of ML in fibromyalgia are reviewed.

### 3.2. Treatment Patterns

AI/ML/DL is opening the door to identify effective treatment options and better outcomes by predicting which treatment protocols are likely to succeed based on patient characteristics, comorbid conditions, and treatment rationales. Recent studies show that different approaches to cluster and subgroup analysis can support more effective treatment choices to treat difficult conditions, as illustrated by overactive bladder [38] or erectile dysfunction (ED) [39]. In particular, researchers identified natural clusters of male characteristics per country, quantified ED dynamics in these profiles and compared profiles. Clusters were mainly predicted by unhealthy behaviors, risk factors, and ED, regardless of positive health characteristics and behaviors. Subgroups of men with heightened ED risk factors were identified for precision medicine for optimal targeted therapies [40]. These examples in noncommunicable diseases (NCDs) show a range of possibilities for making more effective treatment decisions and better managing patient treatment over the course of the disease.

### 3.3. Disease Management

Digital health management has offered long-held hope for extending clinical resources in understanding and managing diseases by virtually connecting patients and healthcare providers through digital technology, such as mobile applications in a bring-your-own-device (BYOD) setting [9,14,41,42]. Data from personal devices can be gathered to support just-in-time adaptive interventions and health behaviors. Such digital tools with usability can help patients receive personalized support and engage with health care providers. 

## 4. Data Volume

Approaches are promising to generate insights from large-scale and high-volume big data, such as those in the form of RWD [26]. There are a set of characteristics needed for trustworthy AI, including “accuracy, explainability and interpretability, privacy, reliability, robustness, safety, and security resilience—and that harmful biases are mitigated or controlled” [43]. However, limited data that do not well represent the populations of interest likely lead to biased models and conclusions since patient diversity might be lacking in historical trials [44,45], which could be due to various social determinants of health (SDOH) [27]. However, it is difficult to achieve without sufficiently large volume of data.

Clinical decision support (CDS) may be adopted early during the clinical evaluation stage [15,46,47]. Increasingly, AI/ML/DL are used to enhance disease understanding and the effectiveness of their therapies. At present, biopharmaceutical companies may face significant barriers in terms of accessing comprehensive and timely patient data due to the siloed nature of systems in terms of interoperability issues. Machine learning tools tend to require large datasets to generate useful results, which would be challenging to the biopharmaceutical companies, as they are mainly focused on RCT data in a much smaller volume or speed. While big data would allow for training, data scientists may apply newer techniques with fewer data points to mine and transfer them [48], despite training on limited labeled information in the data [49,50]. Models for ML can be trained with small datasets using few-shot and n-shot approaches [51,52]. Few-shot learning has the potential to help clean and label datasets, as well as generate more data. This ability to learn with limited labeled data could help re-evaluate unusable data. Few-shot approaches reduce the need to amass a large volume of the right data and to invest in the computer to train a model on those datasets. Zero-shot techniques have the ability to learn from related data or from descriptions of data, rather than designated datasets [52]. These training models generate results derived from limited data may be helpful but may still lack the generalizability and representativeness, which big data would have the advantage of. Thus, biopharmaceutical companies are tailoring their strategies to harness and maximize the values of data, especially in the form of RWD besides RCT data [53,54,55,56,57]. Even with smaller datasets becoming more useful, data sources may undergo standardization, which may be critical for those generated from disparate systems. Common data models (CDMs) may be used to solve the need for a standard format [16].

## 5. Patient Health Information Protection

Laws and regulations have been established over the privacy of protected health information (PHI) [23]. Data privacy protections become critical [20,21,22,23], and data-sharing practices, e.g., cross-Atlantic collaborations, must carefully regard this privacy protection [58,59,60]. Organizations may consider a risk-based approach that goes beyond simple masking techniques in order to produce a high-quality dataset that meets their specific needs for secondary use. These approaches use ML to determine the likelihood of patient re-identification, thus preserving as many critical data elements as possible to support rich insight while still ensuring compliance.

## 6. Use-Case Examples

Biopharmaceutical companies have multiple use-case examples found in the public domain that focused primarily in the following areas: drug discovery and development, clinical trials, drug manufacturing, and patient care.

### 6.1. AI Adoptions

There are several existing use-case examples on the applications of digital endpoints via crowdsourcing from biopharmaceutical study sponsors, which have been collected via crowdsourcing [61]. In addition, the FDA has showcased 90 successful examples of RWE used in medical devices [62,63]. According to the FDA, there were 18 (20%) premarket notification (510[k]) submissions; 14 (15.6%) de novo classification requests; 2 (2.2%) humanitarian device exemptions (HDE) applications; 20 (22.2%) premarket approval (PMA) original applications; 37 (41.1%) PMA panel track supplements. A set of commonly used ML algorithms, including supervised and unsupervised learning methods, has been provided [10].

According to the Deloitte’s 2022 RWE benchmark survey among 17 biopharmaceutical executives, “AI/ML workbench” has been used by 41% of the companies, while 47% plan to develop such a capability [56]. 

There are multiple examples of applications using AI by a number of pharma companies, focusing primarily in the following areas, including drug discovery and development, clinical trials, drug manufacturing, and patient care [64,65,66,67,68,69,70,71,72,73] (Table 2). The potentials of such innovations through AI/ML/DL can be multifold [74,75,76,77,78].

The top three purposes for AI in RWD via use cases are to “enable a data-driven understanding of disease progression for populations of interest”, “analyze subpopulations to understand patient behaviors (e.g., switching patterns, adherence)”, and “segment patients based on disease characteristics and health outcomes to match them to trials”. An additional seven benefits are also summarized by Deloitte [56].

### 6.2. ML for Fibromyalgia and Pain

Magnetic resonance imaging has been used to distinguish the brain scans of individuals with and without fibromyalgia [79]. Characterization of individuals with fibromyalgia was based on brain futures. Hierarchical clustering was used in another study to evaluate chronic pain subgroups [80]. In addition, researchers found that ML could diagnose fibromyalgia with nearly 90% accuracy using a composition of the microbiome [81]. Nearly 20 bacterial species were identified to increase or decrease among patients with fibromyalgia. Furthermore, an ML study involving neural networks indicated the best immune biomarker for diagnosis [82]. Researchers analyzed a measure to assess alexithymia among fibromyalgia patients [83]. Moreover, time-series analysis was conducted for predictive analysis of pain among patients with painful diabetic peripheral neuropathy [84]. 

## 7. AI and COVID-19

The COVID-19 pandemic urgently demanded an accelerated pace in diagnostic, prevention, and treatment breakthroughs. However, limited data initially made it challenging for AI/ML/DL predictive algorithms to be developed and deployed. Open databases, such as the COVID-19 Open Research Dataset Challenge (CORD-19) [85], facilitated the use of text analysis to mine the literature, and consequently knowledge of the virus and its mechanisms expanded. A confluence and relationship between patient characteristics and comorbid conditions, such as NCDs, and the burden of this infectious disease helped outcome predictions and disease management [86,87,88]. 

**Figure 2:**
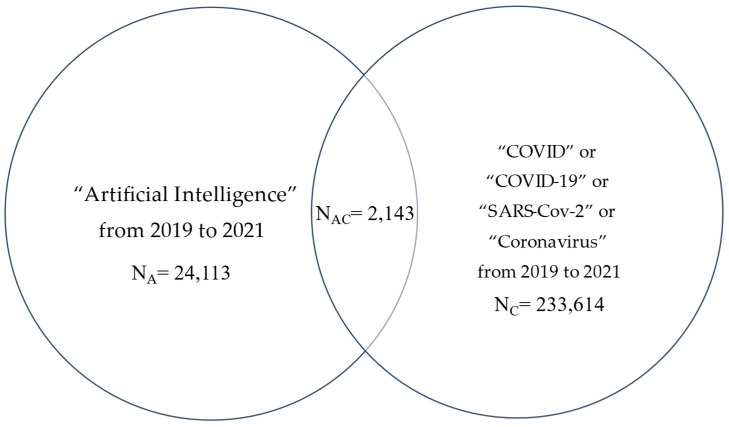
Number of literatures on AI and COVID-19 from 2019 to 2021.

We obtained several numbers of PubMed-listed articles [32]. By using search terms and limiting publications from 2019 to 2021 inclusive and Boolean operators, we focused on: String A alone; String C alone; Strings AC = A and C, where A = “Artificial Intelligence” with nearly 35,000 articles, and C = (“SARS-CoV-2” or “COVID” or “COVID-19” or “Coronavirus”) with nearly 320,000 articles. In addition, a Venn diagram was used to demonstrate the overlap of AC with over 3000 articles in three years (Figure 2).

**Figure 3:**
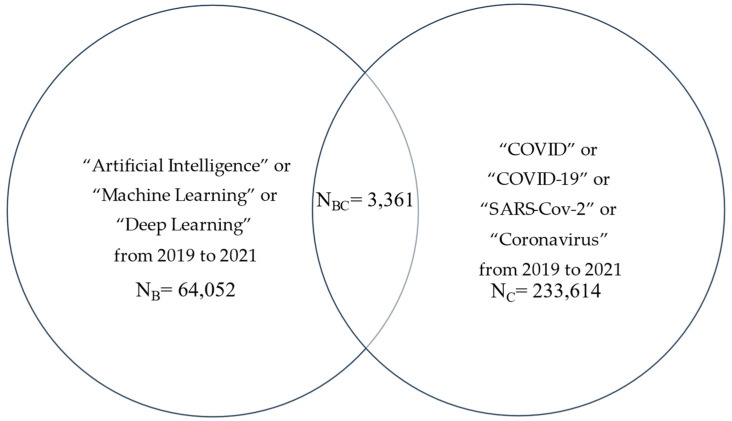
Number of literatures on AI/ML/DL and COVID-19 from 2019 to 2021.

Similarly, we expanded the literature search using: String B alone; String C alone; String BC = B and C, where B = (“Artificial Intelligence” or “Machine Learning” or “Deep Learning”) to represent data science with over 64,000 articles, and again C = (“SARS-CoV-2” or “COVID” or “COVID-19” or “Coronavirus”) with 320,000 articles. The overlap of BC also yielded over 3000 articles during the same period (Figure 3).

Due to the impact of the COVID-19 pandemic on global health, there was an explosion of publications, most of them published in pre-print servers to be disseminated in a timely fashion. For example, one of these articles showed the relationship between natural language and viral evolution [36]. Additional pandemic-specific articles cover a wide range of topics from contact tracing, detection, diagnosis, to drug repurposing (e.g., [89,90,91,92]).

## 8. Conclusions

Biopharma companies have placed a significant commitment in leveraging ML through the use of RWD besides RCTs [53,54,55,56]. The need to address the COVID-19 pandemic over the last several years has shown the need for advances in AI/ML/DL capabilities. There remains a need for agreed regulatory approaches, operating models, and governance, as well as data science talents who understand end-to-end R&D process and health technology assessments in order to enable a much wider spectrum of successful use-case applications. 

Developing these capabilities will be a core element in future patient-centric approaches, as one of the top 10 priorities for health economics and outcomes research in 2022 to 2023 [57]. Significant efforts and extensive strategies are needed for biopharmaceutical industries to conduct such activities. As shown in the literature, AI/ML/DL can make a meaningful difference and provide data-driven approaches for stakeholders across the healthcare ecosystem. Such an intersection between data science, AI/ML/DL algorithms, and digital health innovation also presents opportunities for the biopharmaceutical industry, and more broadly, the healthcare industry, to enhance and improve patient care, although with caution on how explainable AI may limit the benefits of black-box ML/DL algorithms [9,93,94,95,96,97,98]. Finally, it is important to emphasize a holistic approach to AI [99], as in the recent AI Bill of Rights in the US [100].

## Figures and Tables

**Table 1:** Key abbreviations in health data analytics via AI, ML and DL.

Abbreviation	Terminology	Source	Reference
AI	Artificial Intelligence	FDA	[13]
BYOD	Bring Your Own Device	EMA	[14]
CDS	Clinical Decision Support	FDA	[15]
CDM	Common Data Model	National Coordinator for Health Information Technology (HealthIT.gov, accessed on 6 October 2022)	[16]
DL	Deep Learning	FDA	[17]
DTC	Decentralized Clinical Trial	FDA	[18]
DTx	Digital Therapeutics	EU	[19]
GDPR	General Data Protection Regulation	GDPR.EU	[20]
HIPAA	The Health Insurance Portability and Accountability Act of 1996	U.S. Department of Health and Health Services (HHS)	[21]
ML	Machine Learning	FDA	[17]
PCT	Pragmatic Clinical Trial	National Institute of Aging	[22]
PHI	Protected Health Information	HHS.gov	[23]
R&D	Research and Development	Congressional Budget Office	[24]
RCT	Randomized Controlled Trial	National Cancer Institute	[25]
RWD	Real-World Data	FDA	[26]
RWE	Real-World Evidence	FDA	[26]
SDOH	Social Determinants of Health	HHS	[27]

**Table 2:** Examples of Ten Biopharmaceutical Companies’ Harnessing AI/ML/DL via Publicly Available Sources.

Example	Organization	Purpose	Project	Reference
1	AbbVie	Compound Screening	“ChemBeads: Improving Artificial Intelligence Through Human Ingenuty.”	[64]
2	Amgen	Drug Discovery and Development	“AI & Data Science: Opening Up Vast New Frontiers in Drug Discovery and Development.”	[65]
3	AstraZeneca	Drug Discovery and Delivery	“Data Science & Artificial Intelligence: Unlocking New Science Insights.”	[66]
5	GSK (with Massachusetts Institute of Technology; MIT)	Manufacturing	“GSK Manufacturing Initiative.”	[67]
6	Johnson & Johnson	Drug Discovery	“Can Artificial Intelligence Change How We Discover Drugs?”	[68]
7	Merck	Drug Discovery and Development	“Merck Announces the Launch of the Merck Digital Sciences Studio to Help Healthcare Startups Quickly Bring their Innovations to Market.”	[69]
8	Novartis	Disease Diagnosis	“AI-powered Diagnostic Tool to Aid in the Early Detection of Leprosy.”	[70]
8	Pfizer (with CytoReason)	Drug Discovery and Development	“CytoReason Announces Expanded Collaboration Deal with Pfizer to Deliver AI for Drug Discovery and Development.”	[71]
9	Roche	Biomarker Evaluation	“Roche Announces the Release of Its Newest Artificial Intelligence (AI) Based Digital Pathology Algorithms to Aid Pathologists in Evaluation of Breast Cancer Markers, Ki-67, ER and PR.”	[72]
10	Takeda (with MIT)	Human Health and Drug Development	“MIT-Takeda Program Launches: Research Projects Will Harness the Power of Artificial Intelligence to Positively Impact Human Health.”	[73]

## Data Availability

Not applicable.

## Disclaimer

K.H.Z. and L.J.Z. are employees of Viatris Inc., merged between the Upjohn Division of Pfizer Inc. and Mylan Inc. The views expressed are their own and do not necessarily represent those of their employers. This article was based on an early version of a newsletter article, entitled “Enhanced patient-centricity: how the biopharmaceutical industry is optimizing patient care through AI/ML/DL” published in *PharmaVoice* and received a permission from its editorial office for its adaptation for peer-reviewed journal submission. However, this earlier version is unavailable in any public domain, and therefore K.H.Z. and L.J.Z. have revised critically since that prior newsletter article.
